# Xiaoaiping injection as adjunct therapy for patients with advanced esophageal carcinoma: A protocol for a systematic review and meta-analysis

**DOI:** 10.1097/MD.0000000000020984

**Published:** 2020-06-26

**Authors:** Zhen Liu, Yanling Dong, Meili Zhu, Ying Mu, Lemei Chen

**Affiliations:** a Department of Neurology; b Department of Infectious Diseases, People's Hospital of Weifang Binhai Economic and Technological Development Zone, Weifang; c Department of Gastroenterology, Liaocheng People's Hospital, Liaocheng; d Department of General Surgery, People's Hospital of Weifang Binhai Economic and Technological Development Zone, Weifang, Shandong Province, China.

**Keywords:** efficacy, esophageal carcinoma, meta–analysis, safety, Xiaoaiping injection

## Abstract

**Background:**

Esophageal carcinoma (EC) is one of the worst malignant digestive neoplasms with a strong tendency of invasion and metastasis. Despite the improvement of diagnostic and therapeutic methods in the past decades, the prognosis of EC remains unsatisfactory. Xiaoaiping injection (XAPI), a famous traditional Chinese herbal medicine, has been widely applied as a promising adjunctive drug for EC. However, the exact effects and safety of XAPI have yet to be systematically investigated. We aimed to summarize the efficacy and safety of XAPI for the treatment of advanced EC through the meta-analysis, in order to provide scientific reference for the design of future clinical trials.

**Methods:**

Relevant randomized controlled trials (RCTs) were searched from Cochrane Library, PubMed, Google Scholar, Web of Science, Excerpt Medica Database, Medline, China National Knowledge Infrastructure, Chinese Biomedical Literature Database, China Scientific Journal Database and Wanfang Database. Papers in English or Chinese published from January 2000 to May 2020 will be included without any restrictions.

Study selection and data extraction will be performed independently by 2 investigators. The clinical outcomes including overall response rate, complete response rate, overall survival, Disease-free survival, quality of life, immune function and adverse events, were systematically evaluated. Review Manager 5.3 and Stata 14.0 were used for data analysis, and the quality of the studies was also evaluated.

**Results:**

The results of this study will be published in a peer-reviewed journal, and provide more evidence-based guidance in clinical practice.

**Conclusion:**

Our study will draw an objective conclusion of the effects of XAPI combined with conventional treatment for advanced EC and provide a helpful evidence for clinicians to formulate the best postoperative adjuvant treatment strategy for EC patients.

**INPLASY registration number:**

INPLASY202050094.

## 1 Introduction

Esophageal carcinoma (EC) is the ninth most commonly diagnosed cancer and the fifth leading cause of cancer-related deaths.^[1,2]^ It caused 357,190 deaths worldwide only in 2018.^[1,2]^ The incidence of EC has increased exponentially over the past few decades, with about 400,000 new cases per year.^[1,2]^ Among them, 50% of newly diagnosed patients were occurred in China.^[3,4]^ The etiology of EC is still unclear, with possible factors including dietary habits, environmental factors, work pressure, genetic factors and so on.^[5]^ EC is also one of the worst malignant digestive neoplasms with a strong tendency of invasion and metastasis.^[6,7]^ Despite the improvement of diagnostic and therapeutic methods in the past decades, the prognosis of EC remains unsatisfactory.^[6–8]^ Most EC patients already have advanced or metastatic lesions when diagnosed, due to the lack of noticeable clinical symptoms at its early stage.^[7]^ The 5-year survival rate of stage III EC patients was about 20%, while that of stage IV patients was reduced to 10%.^[7,9]^ Currently, the clinical treatment of EC mainly includes radiotherapy, chemotherapy, surgical resection alone or combined strategy.^[7,8,10–12]^ However, their applications are limited by failing to thoroughly eliminate tumor cells, drug resistance and other adverse effects.^[8,12]^ Therefore, exploring new regimens with better tolerance and lower toxicity for patients with esophageal cancer are urgently required.

Traditional Chinese Medicine has been used as an adjunct treatment for alleviating the side effects of radiochemotherapy and for improving the quality of life (QoL) of cancer patients.^[12–19]^ Some researchers indicated that the combination of Chinese and Western medicine for EC may be the potential trend of clinical treatment development in future.^[12,16–19]^ The monomer compounds obtained from medicinal herbs has exhibit potential anti-cancer activity against various type tumors including EC.^[12,16–20]^ Xiaoaiping injection (XAPI) is a famous traditional Chinese herbal medicine extracted from the root of Marsdenia tenacissima (Tong Guan Teng or Tong Guang Teng), containing flavonoids, polysaccharides, steroidal saponins, alkaloids, triterpenes and other chemical constituents, which has been reported to have antitumor effect.^[21–24]^ The anti-tumor pharmaological effects of XAPI is mainly includes the following 2 aspects:

1. direct anti-tumor effects, such as inhibiting tumor cell proliferation and invasion, inducing cell cycle arrest and apoptosis, and inhibiting tumor cell angiogenesis;^[21,24–26]^ and
2. enhance the antitumor effect by increasing the sensitivity of the tumor cells to radiochemotherapy.^[24,27,28]^

**Figure 1:**
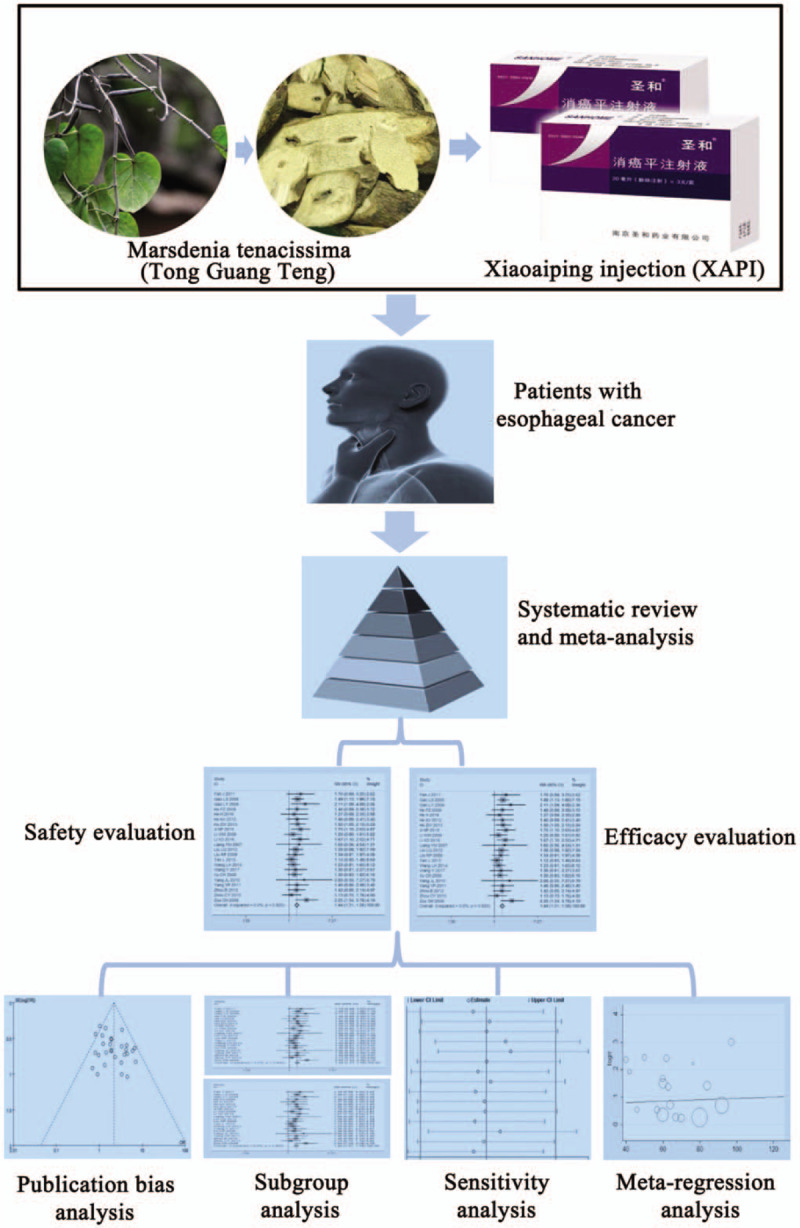
Work flow of the present study.

Clinical trials have indicated that the combination of XAPI and classic radiochemotherapy not only exerts an enhanced therapeutic effect against EC, but also improve QoL and immune function, and reduce the incidence of side effects caused by radiochemotherapy.^[25,29]^ Despite the intensive clinical studies, its clinical efficacy was still not well established and recognized. We are prepared to summarize the efficacy and adverse events of XAPI treatment of EC at advanced stages through the meta-analysis, in order to provide scientific reference for the design of future clinical trials (Fig. 1).

## 2 Objective

A systematic review and meta-analysis will be performed to systematically evaluate the efficacy and safety of XAPI adjuvant therapy combined with conventional treatment for advanced EC.

## 3 Methods

The protocol of our meta-analysis will be reported according to Preferred Reporting Items for Systematic Review and Meta-Analysis Protocols (PRISMA-P) guidelines.^[30]^ Our protocol has been registered on the International Platform of Registered Systematic Review and Meta-Analysis Protocols (INPLASY). The registration number was INPLASY202050094 (DOI number is 10.37766/inplasy2020.5.0094, https://inplasy.com/inplasy-2020-5-0094/). This meta-analysis is a secondary research which based on some previously published data. Therefore, the ethical approval or informed consent was not required in this study.

### 3.1 Eligibility criteria

#### 3.1.1 Types of studies

All available randomized controlled trials (RCTs) or quasi-RCTs, and high-quality prospective cohort studies that investigated the efficacy and safety of XAPI-mediated therapy in patients diagnosed with advanced EC will be included in this systematic review.

#### 3.1.2 Types of participants

Patients must be cytologically or pathologically confirmed as having EC at a clinically advanced stage. There will be no restrictions regarding age, gender, racial, region, education and economic status. Patients with other malignancies or non-primary EC are not included.

#### 3.1.3 Types of interventions

In the experimental group, advanced EC patients must be treated with conventional treatment (including chemotherapy, radiotherapy, and targeted therapy) combined with XAPI mediated therapy.

#### 3.1.4 Comparator

In the control group, EC patient treated with the same conventional treatment as intervention group in the same original study.

#### 3.1.5 Exclusion criteria

Articles without sufficient available data, non-comparative studies, non-peer reviewed articles, meta-analysis, literature reviews, case reports, case series, meeting abstracts, animal studies, letter to the editor, commentaries, editorials, and other unrelated studies will be excluded from analysis.

#### 3.1.6 Types of outcome measures

##### 3.1.6.1 Primary outcomes

The primary outcomes will be the therapeutic effects of treatment according to Response Evaluation Criteria in Solid Tumors 1.1 (RECIST Criteria 1.1).^[31]^

1. Overall response rate (ORR);
2. Overall survival (OS, which is defined as the time from the date of randomization to death from any cause);
3. Disease-free survival (DFS, which is the time from date of random assignment to date of recurrence or death).

##### 3.1.6.2 Secondary outcomes

Secondary outcomes will include:

1. QoL as evaluated by Karnofsky score;
2. Immune function;
3. Treatment–related adverse effects.

### 3.2 Information sources

Electronic databases including Cochrane Library, PubMed, Google Scholar, Web of Science (WOS), Excerpt Medica Database (Embase), Medline, China National Knowledge Infrastructure (CNKI), Chinese Biomedical Literature Database (CBM), China Scientific Journal Database (VIP) and Wanfang Database will be systematically searched for eligible studies from January 2000 to May 2020. Language is limited with English and Chinese.

### 3.3 Search strategy

To perform a comprehensive and focused search, experienced systematic review researchers will be invited to develop a search strategy. The plan searched terms are as follows: “esophageal cancers” or “esophageal neoplasm” or “esophageal carcinoma” or “esophageal tumor” or “shiguanai” or “shiguanzhongliu” or “EC” and “Xiaoaiping injection” or “XAP injection” or “XAPI” or “Tongguanteng” or “Tongguanteng extract” or “Tongguangteng” or “Tongguangteng extract” or “Marsdenia tenacissima” or “Marsdenia tenacissima extract” or “MTE” et al. An example of search strategy for PubMed database shown in Table 1 will be modified and used for the other databases.

**Table 1:**
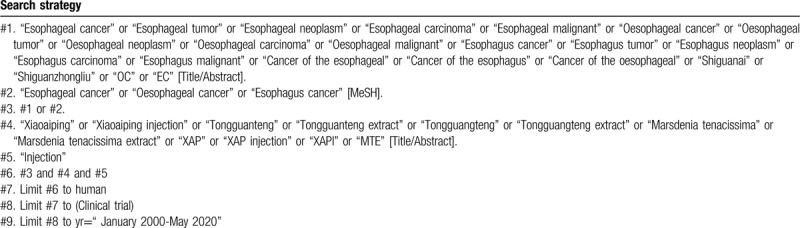
Searching strategy in PubMed.

### 3.4 Data collection and analysis

We will adopt the measures described in the Cochrane Handbook for Systematic Reviews of Interventions to pool the evidence.^[32]^

#### 3.4.1 Study selection and management

**Figure 2:**
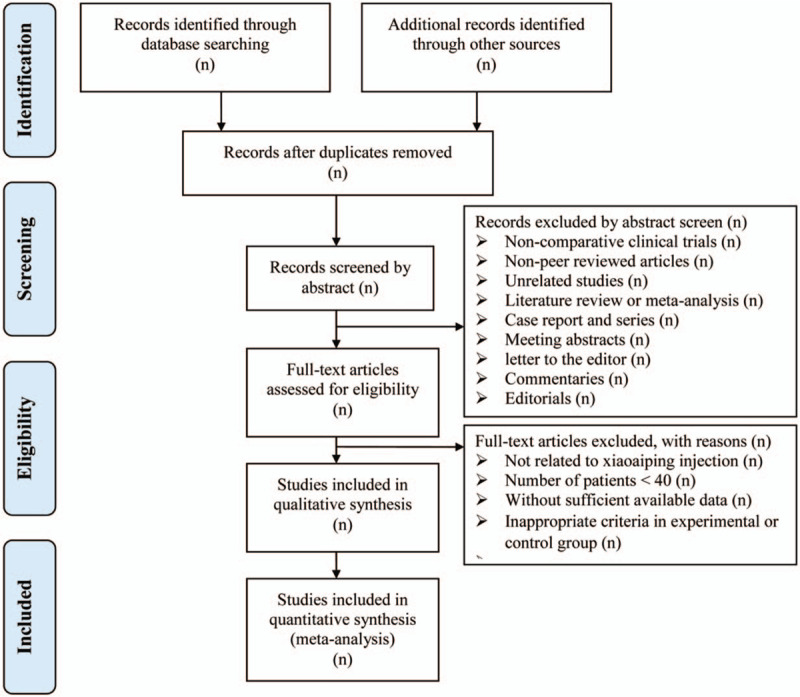
Study selection process for the meta-analysis.

Two authors (Zhen Liu and Yanling Dong) will be reviewed independently to identify potential trials by assessing the titles and abstracts and identify whether the trials meet the inclusion criteria as designed and described in this protocol. Two reviewers (Zhen Liu and Yanling Dong) will in duplicate and independently screen the full text of all potential eligible studies to exclude irrelevant studies or determine eligibility. The 2 reviewers (Zhen Liu and Yanling Dong) will list all the studies included and document the primary reasons of exclusion for studies that do not conform to the inclusion criteria. Disagreements between the 2 authors will be resolved by discussing with the third author (Meili Zhu), if necessary, consulting with the fourth author (Ying Mu). A PRISMA-compliant flow chart (Fig. 2) will be used to describe the selection process of eligible literatures. Endnote X7 software will be used for literature managing and records searching.

#### 3.4.2 Data extraction and management

Two reviewers (Zhen Liu and Yanling Dong) will be responsible for the data extraction independently according to the Cochrane Handbook for Systematic Reviews of Intervention.

The following data will be extracted from eligible literatures:

1. Study characteristics and methodology: country of study, the first author, year of publication, study design, randomization, periods of data collection, total duration of study, follow-up duration, and withdrawals, et al.
2. Participant characteristics: sample size, tumor stage (staging of the tumor according to the AJCC TNM classification for esophageal cancer), age, gender, ethnicity, pathology diagnosis, pathologic tumor size, inclusion, and exclusion criteria, etc.
3. Interventions: therapeutic means, manufacturer of the drugs, dosage of XAPI, administration route and cycles, duration of treatment and follow-up time, et al.
4. Outcome and other data: ORR, OS, DFS, QoL, immune function and adverse effects, et al. For survival outcomes, Hazard ratios (HRs) with corresponding 95% confidence intervals (CIs) will be extracted from trials or be estimated from Kaplan–Meier survival curves by established methods.^[33]^

Dealing with missing data: we will attempt to contact the authors to request the missing or incomplete data. If those relevant data are not acquired, they will be excluded from the analysis. Any disagreements will be resolved by discussion, and a third reviewer (Meili Zhu) will make the final decision. Excluded studies and the reasons for exclusion will be listed in a table.

### 3.5 Assessment of risk of bias

The quality of the included RCTs will be assessed independently by 2 investigators (Zhen Liu and Yanling Dong) in terms of random sequence generation (selection bias), allocation concealment (selection bias), blinding of participants and personnel (performance bias), blinding of outcome assessment (detection bias), incomplete outcome data (attrition bias), selective outcome reporting (reporting bias), and other bias, according to the guidance of the Cochrane Handbook for Systematic Review of Interventions.^[32,34]^ Evidence quality will be classified as low risk, high risk, or unclear risk of bias in accordance with the criteria of the risk of bias judgment. The risk of bias judgments across different studies for each of the domains listed will be summarized. EPOC guidelines will be used to assess the risks of non-RCTs.^[35]^ Any disagreements will be resolved via discussion with a third researcher (Meili Zhu).

### 3.6 Data synthesis

Data from studies judged to be clinically homogeneous will be pooled using Review Manager 5.3 (Nordic Cochran Centre, Copenhagen, Denmark) and Stata 14.0 (Stata Corp., College Station, TX, USA) statistical software. Heterogeneity between studies will be assessed using the Cochrans Q and Higgins *I*^*2*^ statistic. *P* < .1 for the Chi^2^ statistic or an *I*^*2*^ > 50% will be considered as showing considerable heterogeneity.^[36]^ A fixed effect model will be used to calculate the outcomes when statistical heterogeneity is absent; otherwise, the random effects model was considered according to the DerSimonian and Laird method.^[37]^ The Mantel–Haenszel method will be applied for pooling of dichotomous data and results will be presented as relative risk (RR) with their 95% confidence intervals (CIs). Inverse variance method will be used for pooling of continuous data and results will be presented as standardized mean difference (SMD) with their 95% CIs. A two-tailed *P* value <.05 was considered statistically significant.

### 3.7 Subgroup and meta-regression analysis

If the data are available and sufficient, subgroup and meta-regression analysis will be conducted to explore the source of heterogeneity with respect to age, gender, tumor stage, region, course of treatment and therapeutic regimens.

### 3.8 Sensitivity analysis

Sensitivity analysis will be conducted to assess the reliability and robustness of the aggregation results via eliminating trials with high bias risk. A summary table will report the results of the sensitivity analyses.

### 3.9 Publication bias analysis

We will detect publication biases and poor methodological quality of small studies using funnel plots if 10 or more studies are included in the meta-analysis. Begg and Egger regression test will be utilized to detect the funnel plot asymmetry.^[38–40]^ If reporting bias is suspected, we will consult the study author to get more information. If publication bias existed, a trim-and-fill method should be applied to coordinate the estimates from unpublished studies, and the adjusted results were compared with the original pooled RR.^[41,42]^

### 3.10 Evidence evaluation

The evidence grade will be determined by using the guidelines of the Grading of Recommendations, Assessment, Development, and Evaluation (GRADE). The quality of all evidence will be evaluated as 4 levels (high, moderate, low, and very low).^[32]^

#### 3.11 Dissemination plans

We will disseminate the results of this systematic review by publishing the manuscript in a peer-reviewed journal or presenting the findings at a relevant conference.

## 4 Discussion

EC is a highly malignant tumor, although there is a variety of advanced treatment methods combined with surgical treatment, but the patient prognosis is very poor.^[6,7,12]^ Therefore, therapies that could significantly improve OS and have fewer side effects are what we need to pursue now.^[12]^ Traditional Chinese Medicine is a prominent complementary and alternative medicine for cancer treatment.^[13,15]^ Currently, it has reported that medicinal herbs have a unique advantage in EC therapy by inhibiting the growth of cancer cells, mitigating the progress of the disease, enhancing immunity, decreasing cancer relapses and metastases, increasing 5-year survival rate.^[12,16–18,20]^ XAPI, a drug that is mainly composed of the Chinese herb Marsdeniae tenacissimae was manufactured by Tonghua Jinma and Nanjing Shenghe Pharmaceutical Co., Ltd. It have been approved by Chinese State Food and Drug Administration (SFDA), and granted the Manufacturing Approve Number accordingly (Z20025869 and Z20025868).^[24]^ It has been applied alone or combined with chemotherapy or radiotherapy to treat various malignant tumors in China.

### 4.1 Strengths and limitations of this study

Even though there was statistical analysis of published clinical trials, the exact therapeutic effects of XAPI mediated therapy for EC were still not systematically investigated. Thus, in-depth knowledge of the efficacy and safety of XAPI is needed. We will conduct a systematic, comprehensive and objective evaluation of XAPI-based adjuvant therapy. The results of this study will provide a helpful evidence for clinicians to formulate the best postoperative adjuvant treatment strategy for patients with advanced EC, and also provide scientific clues for researchers in this field.

The systematic review will also have some limitations. There may be a language bias with the limitation of English and Chinese studies. In addition, due to the nature of the disease and intervention, large sample clinical trials are not abundant, so we will include some high-quality small sample trials, which may cause high heterogeneity.

## Author contributions

Lemei Chen and Zhen Liu conceived the concept and designed the study protocol. Zhen Liu, Yanling Dong and Lemei Chen tested the feasibility of the study. Zhen Liu, Yanling Dong and Meili Zhu wrote the manuscript. Zhen Liu, Lemei Chen and Ying Mu provided methodological advice, polished and revised the manuscript. All authors approved the final version of the manuscript.

**Conceptualization:** Lemei Chen and Zhen Liu.

**Funding acquisition:** Ying Mu.

**Investigation:** Zhen Liu, Yanling Dong and Meili Zhu.

**Methodology:** Zhen Liu, Yanling Dong, Meili Zhu, Lemei Chen and Ying Mu.

**Project administration:** Zhen Liu and Lemei Chen.

**Supervision:** Lemei Chen and Zhen Liu.

**Writing – original draft:** Zhen Liu, Yanling Dong and Meili Zhu.

**Writing – review & editing:** Zhen Liu, Lemei Chen and Ying Mu.

## References

[1] Bray F Ferlay J Soerjomataram I Global cancer statistics 2018: GLOBOCAN estimates of incidence and mortality worldwide for 36 cancers in 185 countries. CA Cancer J Clin 2018;68:394–424. 30207593 10.3322/caac.21492

[2] Ferlay J Colombet M Soerjomataram I Estimating the global cancer incidence and mortality in 2018: GLOBOCAN sources and methods. Int J Cancer 2019;144:1941–53. 30350310 10.1002/ijc.31937

[3] Chen W Zheng R Baade PD Cancer statistics in China, 2015. CA Cancer J Clin 2016;66:115–32. 26808342 10.3322/caac.21338

[4] Feng RM Zong YN Cao SM Current cancer situation in China: good or bad news from the 2018 Global Cancer Statistics? Cancer Commun 2019;39:22. 10.1186/s40880-019-0368-6 PMC6487510 31030667

[5] Huang FL Yu SJ Esophageal cancer: risk factors, genetic association, and treatment. Asian J Surg 2018;41:210–5. 27986415 10.1016/j.asjsur.2016.10.005

[6] Wang W Xing D Song Y Effects of S-1 combined with radiotherapy in the treatment of advanced esophageal cancer: a systematic review and meta-analysis protocol. Medicine 2018;97:e0164. 29561425 10.1097/MD.0000000000010164 PMC5895356

[7] Chai T Shen Z Zhang P Postoperative adjuvant therapy for resectable esophageal cancer: A protocol of a systematic review and meta-analysis. Medicine 2019;98:e15485. 31096448 10.1097/MD.0000000000015485 PMC6531204

[8] Liu Y Mu Y Zhang A Cytokine-induced killer cells/dendritic cells and cytokine-induced killer cells immunotherapy for the treatment of esophageal cancer in China: a meta-analysis. Onco Targets Ther 2017;10:1897–908. 28408841 10.2147/OTT.S132507 PMC5384723

[9] Ando N Ozawa S Kitagawa Y Improvement in the results of surgical treatment of advanced squamous esophageal carcinoma during 15 consecutive years. Ann Surg 2000;232:225–32. 10903602 10.1097/00000658-200008000-00013 PMC1421135

[10] Bollschweiler E Plum P Mönig SP Current and future treatment options for esophageal cancer in the elderly. Expert Opin Pharmacother 2017;18:1001–10. 28540761 10.1080/14656566.2017.1334764

[11] Ku GY Current treatment of esophageal cancer and promising clinical trials underway. Oncology 2019;33:110–2. 30866034

[12] Ying J Zhang M Qiu X The potential of herb medicines in the treatment of esophageal cancer. Biomed Pharmacother 2018;103:381–90. 29674273 10.1016/j.biopha.2018.04.088

[13] Wang Z Qi F Cui Y An update on Chinese herbal medicines as adjuvant treatment of anticancer therapeutics. Biosci Trends 2018;12:220–39. 30012913 10.5582/bst.2018.01144

[14] Lin AX Chan G Hu Y Internationalization of traditional Chinese medicine: current international market, internationalization challenges and prospective suggestions. Chin Med 2018;13:9. 29449877 10.1186/s13020-018-0167-z PMC5807832

[15] Liu J Wang S Zhang Y Traditional Chinese medicine and cancer: History, present situation, and development. Thorac Cancer 2015;6:561–9. 26445604 10.1111/1759-7714.12270 PMC4567000

[16] Wu T Yang X Zeng X Traditional Chinese medicinal herbs in the treatment of patients with esophageal cancer: a systematic review. Gastroenterol Clin North Am 2009;38:153–67, x. 19327573 10.1016/j.gtc.2009.01.006

[17] Lu P Liang QD Li R Effect of traditional chinese medicine on survival and quality of life in patients with esophageal carcinoma after esophagectomy. Chin J Integr Med 2006;12:175–9. 17005076 10.1007/BF02836517

[18] Chen X Deng L Jiang X Chinese herbal medicine for oesophageal cancer. Cochrane Database Syst Rev 2016;CD004520. 26799001 10.1002/14651858.CD004520.pub7 PMC10217096

[19] Zhang D Wu J Wang H Systematic review and network meta-analysis comparing Chinese herbal injections with chemotherapy for treating patients with esophageal cancer. J Int Med Res 2020;48: 300060519898336. 10.1177/0300060519898336 PMC7113717 31948305

[20] Zhang YS Shen Q Li J Traditional Chinese medicine targeting apoptotic mechanisms for esophageal cancer therapy. Acta Pharmacol Sin 2016;37:295–302. 26707140 10.1038/aps.2015.116 PMC4775842

[21] Zheng AW Chen YQ Fang J Xiaoaiping combined with cisplatin can inhibit proliferation and invasion and induce cell cycle arrest and apoptosis in human ovarian cancer cell lines. Biomed Pharmacother 2017;89:1172–7. 28320083 10.1016/j.biopha.2017.03.012

[22] Qi S Li X Dong Q Chinese Herbal Medicine (Xiaoaiping) injections for chemotherapy-induced thrombocytopenia: a randomized, controlled, multicenter clinical trial. J Altern Complement Med 2019;25:648–55. 31090434 10.1089/acm.2018.0470 PMC6590720

[23] Li J Zhang Y Liu K Xiaoaiping induces developmental toxicity in zebrafish embryos through activation of ER stress, apoptosis and the Wnt pathway. Front Pharmacol 2018;9:1250. 30459614 10.3389/fphar.2018.01250 PMC6233021

[24] Yu F Li Y Zou J The Chinese herb Xiaoaiping protects against breast cancer chemotherapy-induced alopecia and other side effects: a randomized controlled trial. J Int Med Res 2019;47:2607–14. 31099281 10.1177/0300060519842781 PMC6567696

[25] Fan W Sun L Zhou JQ Marsdenia tenacissima extract induces G0/G1 cell cycle arrest in human esophageal carcinoma cells by inhibiting mitogen-activated protein kinase (MAPK) signaling pathway. Chin J Nat Med 2015;13:428–37. 26073339 10.1016/S1875-5364(15)30036-4

[26] Zheng A Li T Chen Y Inhibitory effect of a Chinese medicine Xiaoaiping combined with cisplatin on the proliferation, invasion and apoptosis in ovarian cancer HO-8910 PM cells in vitro and in vivo. Chin J Oncol 2016;38:11–6. 10.3760/cma.j.issn.0253-3766.2016.01.003 26796800

[27] Ruan LW Deng YC Study on effect of Xiaoaiping in enhancing efficacy of neoadjuvant chemotherapy for breast cancer and its mechanism. Chin J tradit Chin Med 2015;40:749–52. 26137702

[28] Han SY Zhao MB Zhuang GB Marsdenia tenacissima extract restored gefitinib sensitivity in resistant non-small cell lung cancer cells. Lung Cancer 2012;75:30–7. 21757251 10.1016/j.lungcan.2011.06.001

[29] Wang F Fan QX Wang HH Efficacy and safety of Xiaoaiping combined with chemotherapy in the treatment of advanced esophageal cancer. Chin J Oncol 2017;39:453–7. 10.3760/cma.j.issn.0253-3766.2017.06.010 28635236

[30] Shamseer L Moher D Clarke M Preferred reporting items for systematic review and meta-analysis protocols (PRISMA-P) 2015: elaboration and explanation. BMJ 2015;350:g7647. 25555855 10.1136/bmj.g7647

[31] Schwartz LH Litiere S de Vries E RECIST 1.1-Update and clarification: From the RECIST committee. Eur J Cancer 2016;62:132–7. 27189322 10.1016/j.ejca.2016.03.081 PMC5737828

[32] Higgins JP Altman DG Gotzsche PC The Cochrane Collaboration's tool for assessing risk of bias in randomised trials. BMJ 2011;343:d5928. 22008217 10.1136/bmj.d5928 PMC3196245

[33] Tierney JF Stewart LA Ghersi D Practical methods for incorporating summary time-to-event data into meta-analysis. Trials 2007;8:16. 17555582 10.1186/1745-6215-8-16 PMC1920534

[34] Zeng X Zhang Y Kwong JS The methodological quality assessment tools for preclinical and clinical studies, systematic review and meta-analysis, and clinical practice guideline: a systematic review. J Evid Based Med 2015;8:2–10. 25594108 10.1111/jebm.12141

[35] Grimshaw J McAuley LM Bero LA Systematic reviews of the effectiveness of quality improvement strategies and programmes. Qual Saf Health Care 2003;12:298–303. 12897365 10.1136/qhc.12.4.298 PMC1743751

[36] Jackson D White IR Riley RD Quantifying the impact of between-study heterogeneity in multivariate meta-analyses. Stat Med 2012;31:3805–20. 22763950 10.1002/sim.5453 PMC3546377

[37] George BJ Aban IB An application of meta-analysis based on DerSimonian and Laird method. J Nucl Cardiol 2016;23:690–2. 26245193 10.1007/s12350-015-0249-6

[38] Lin L Chu H Quantifying publication bias in meta-analysis. Biometrics 2018;74:785–94. 29141096 10.1111/biom.12817 PMC5953768

[39] Begg CB Mazumdar M Operating characteristics of a rank correlation test for publication bias. Biometrics 1994;50:1088–101. 7786990

[40] Egger M Davey Smith G Schneider M Bias in meta-analysis detected by a simple, graphical test. BMJ 1997;315:629–34. 9310563 10.1136/bmj.315.7109.629 PMC2127453

[41] Shi L Lin L The trim-and-fill method for publication bias: practical guidelines and recommendations based on a large database of meta-analyses. Medicine 2019;98:e15987. 31169736 10.1097/MD.0000000000015987 PMC6571372

[42] Duval S Tweedie R Trim and fill: A simple funnel-plot-based method of testing and adjusting for publication bias in meta-analysis. Biometrics 2000;56:455–63. 10877304 10.1111/j.0006-341x.2000.00455.x

